# Methane production potentials, pathways, and communities of methanogens in vertical sediment profiles of river Sitka

**DOI:** 10.3389/fmicb.2015.00506

**Published:** 2015-05-21

**Authors:** Václav Mach, Martin B. Blaser, Peter Claus, Prem P. Chaudhary, Martin Rulík

**Affiliations:** ^1^Department of Ecology and Environmental Science, Faculty of Science, Palacky UniversityOlomouc, Czech Republic; ^2^Department of Biogeochemistry, Max Planck Institute for Terrestrial MicrobiologyMarburg, Germany

**Keywords:** methane production potential, river sediment, stable carbon isotope, isotope fractionation, depth profile, methyl fluoride, mcrA, T-RFLP

## Abstract

Biological methanogenesis is linked to permanent water logged systems, e.g., rice field soils or lake sediments. In these systems the methanogenic community as well as the pathway of methane formation are well-described. By contrast, the methanogenic potential of river sediments is so far not well-investigated. Therefore, we analyzed (a) the methanogenic potential (incubation experiments), (b) the pathway of methane production (stable carbon isotopes and inhibitor studies), and (c) the methanogenic community composition (terminal restriction length polymorphism of *mcrA*) in depth profiles of sediment cores of River Sitka, Czech Republic. We found two depth-related distinct maxima for the methanogenic potentials (a) The pathway of methane production was dominated by hydrogenotrophic methanogenesis (b) The methanogenic community composition was similar in all depth layers (c) The main TRFs were representative for *Methanosarcina, Methanosaeta, Methanobacterium*, and *Methanomicrobium* species. The isotopic signals of acetate indicated a relative high contribution of chemolithotrophic acetogenesis to the acetate pool.

## Introduction

Biogenic methane production is carried out by highly specialized, oxygen sensitive methanogenic archaea. Usually methanogenesis is therefore restricted to water-logged systems like freshwater sediments, rice field soils or gut systems (Ciais et al., 2014). Rivers as turbulent systems usually have well-aerated water bodies. Hence they are not considered to be an important source of atmospheric methane (Conrad, 2009; Ciais et al., 2014). Even when the methane emission of different fresh water systems (lakes, wetlands etc.,) is compared, the emission rates of rivers are usually low (Table 1).

**Table 1:** **Methane emissions from wetlands**.

**River**	**Methane emission rate (mg CH_4_ m^−2^ h^−1^)**	**References**
River (Nome Creek)^a^	3.5	Crawford et al., 2013
River (Sitka)	0.3–1.6	Hlavacova et al., 2006
Rivers^b^	0.01–6.67	Bastviken et al., 2011
Rivers	10.5	Ortiz-Llorente and Alvarez-Cobelas, 2012
Lakes	18.1	Ortiz-Llorente and Alvarez-Cobelas, 2012
Wetlands	13.6	Ortiz-Llorente and Alvarez-Cobelas, 2012
Estuaries	3.3	Ortiz-Llorente and Alvarez-Cobelas, 2012

a Given as 58.2 nmol CH_4_ m^−2^ s^−1^

b *Calculated from Supplementary Material*.

Methane emission from fresh water systems is usually estimated using the CH_4_ released from open water bodies to the atmosphere. These kind of measurements are showing high spatial fluctuations of methane concentrations (Berger and Heyer, 1989; Lilley et al., 1996; Moura et al., 2008; Wang et al., 2009; Gar'Kusha et al., 2010; Striegl et al., 2012; Musenze et al., 2014) as well as seasonal dynamics (Sanders et al., 2007; Gar'Kusha et al., 2010; Musenze et al., 2014).

However, methane measurements of river water body may not give a conclusive picture of the methanogenic potential of river ecosystems, since the well-aerated water bodies render optimal conditions for methanotrophic bacteria possibly scavenging a large portion of the methane produced in the anoxic parts of the river sediment. Indeed the methane concentrations in the sediment are usually two orders of magnitude higher than in the surface water as can be seen for our study site river Sitka (Hlavacova et al., 2005; Rulík et al., 2013) and several other river sediments (Zaiss, 1981; De Angelis and Scranton, 1993; Trimmer et al., 2009; Gar'Kusha et al., 2010).

In contrast to these *in situ* measurements, which to some extent may be influenced by aerobic methanotrophic activities, the methanogenic production potential of river sediments can be obtained with incubation experiments under strict anoxic conditions in the laboratory. Such experiments have so far only been conducted for mixed top sediments (Jones et al., 1995; Avery and Martens, 1999). In river Sitka preliminary methane production potentials have been estimated with short time incubations under substrate additions (ca 8 μM acetate) (Rulík et al., 2013). Since earlier reports in sediment profiles show vertically dispersed methane concentrations (De Angelis and Scranton, 1993; Schindler and Krabbenhoft, 1998; Gar'Kusha et al., 2010; Chen and Yin, 2013) we decided to test the methanogenic potential of different depth layers of two sediment cores to define whether these differences are due to different methanogenic potentials.

In addition we aimed to differentiate the underlying pathway of methane production. In the well-studied systems (e.g., rice paddies and lake sediments) methane emission can be linked to two dominating processes: acetoclastic (Equation 1) and hydrogenotrophic (Equation 2) methanogenesis:

(1) $${\text{CH}}_{\text{3}}\text{COOH}\to {\text{CO}}_{\text{2}}{\text{ + CH}}_{\text{4}}$$

(2) $${\text{CO}}_{\text{2}}{\text{ + 4H}}_{\text{2}}\to {\text{2H}}_{\text{2}}{\text{O + CH}}_{\text{4}}$$

To distinguish the two dominant methanogenic pathways the natural abundance of stable carbon isotopes can be used if the δ^13^C of methane and of its precursors and the methanogenic fractionation factors are known (Conrad, 2005). The acetoclastic methanogenesis expresses a smaller kinetic isotopic effect (KIE = 1.009−1.027) (Gelwicks et al., 1994; Penning et al., 2006; Goevert and Conrad, 2009) than the hydrogenotrophic methane formation (KIE = 1.045−1.073) (Valentine et al., 2004). The inhibition of acetoclastic methanogenesis by methyl fluoride (CH_3_F) allows quantifying the contribution of both pathways (Janssen and Frenzel, 1997; Conrad et al., 2011).

While the acetoclastic pathway is dominating in e.g., rice paddy soils [up to 67% of methane release (Conrad, 1999)] freshwater sediments and gut environments are dominated by hydrogen driven methanogenesis (Conrad, 1999). The hydrogenotrophic contribution to methane relase for White Oak River sediments was reported to be 37–39% (Avery and Martens, 1999).

As a third aspect we were interested in quantifying the methanogenic community in river sediment profiles and contrast these findings to well-described ecosystems: Lake sediments are dominated by *Methanomicrobiales* and *Methanosaetaceae*. They show gradual vertical changes in methanogenic potential, pathway usage and community composition (Chan et al., 2005). Investigations of mudflat sediments of Yangtze River estuary, China showed a dominance of *Methanomicrobiales* and *Methanosarcinales* (Zeleke et al., 2013). In freshwater systems *Methanomicrobiales* have been shown to increase in relative abundance with depth while *Methanosarcinales*/*Methanosaetaceae* decrease (Chan et al., 2005; Zeleke et al., 2013). Oxygenated upland soils contain a less developed methanogenic community than permanently water-logged systems and are dominated by *Methanocellales* and *Methanosarcinales* (Angel et al., 2012). Rice field soils are generally characterized by the most complex methanogenic community (Chin et al., 1999; Lueders et al., 2001; Ramakrishnan et al., 2001), which has been attributed to the seasonal change of oxic and anoxic conditions. We speculated that the methanogenic community of river sediments will be similar to lake sediments.

In this study, we investigated the methanogenic potential, pathway usage and community structure in river sediment depth profiles. We had three main objectives: (1) we wanted to investigate how the potential methane production rates differ over a vertical profile of two sediment cores in order to validate the potential methane emission rates of river sediment compared to other water logged systems. (2) We wanted to characterize the underlying pathway usage of methane production using the natural stable carbon isotope signals. (3) We were interested in comparing the methanogenic community of river sediments to community profiles of other well-characterized soil systems.

In general we hypothesized that river sediments will share some common features with other freshwater sediments but may also have distinct characteristics due to the water movement and the higher oxygen load of the overlaying water.

## Methods

### Sampling site

The sampling site is situated ca 10 km north of the city Olomouc in an agricultural field area. Stream width ranges between 4 and 6 m during a year. Bottom sediments are composed of clay, sand and gravel having a median grain size of 0.2 mm. More physicochemical parameters (e.g., grain median size, organic carbon content, dissolved O_2_, DOC, interstitial, CH_4_ concentration) in the sediments have already been reported (Buriankova et al., 2012) as locality IV.

### Sediment sampling

Two sediments cores (60 cm deep) were collected using the freeze core method (Bretschko and Klemens, 1986) at morning in April 2012. Sediment cores were split up in layers of 10 cm, sieved with distilled deionized water to 1 mm grain size and stored at 4°C under river water in closed plastic jars.

### Incubation experiments

For determining the methanogenic potential of sediment and carbon isotopic composition of methane and carbon dioxide, the samples were incubated in triplicates under wet anoxic conditions: 5 g of wet sediment samples were supplemented with 2 ml of distilled water and placed in 27-ml pressure tubes, closed with butyl rubber stoppers and incubated under N_2_ at 25°C; if needed 3% (v/v of the headspace) methyl fluoride (CH_3_F) was added to specifically inhibit acetoclastic methanogenesis (Janssen and Frenzel, 1997; Conrad and Klose, 1999). Gas subsamples (0.1–0.4 ml) were taken repeatedly from the headspace using a gas-tight syringe (VICI) and analyzed for concentration and δ^13^C of CH_4_ and CO_2_. Methane production potentials were calculated as slope of the methane concentration over time using at least three data points during the linear phase of methane release. The production potentials are given in nmol CH_4_ per gram dry weight (DW) per day. The water content of fresh samples was approximately 24.6% ± 4.

At the end of the incubation, the vials were sacrificed, sediments were centrifuged and the supernatants were filtered through 0.2 − μm polytetrafluoroethylene (PTFE) membrane filters and stored at −20°C for later analysis of concentration and δ^13^C of acetate (and other fatty acids).

### *In-situ* gas measurements

At morning time in October 2012, sampling of gas ebullition from river sediments was carried out at the same stream stretch from where sediment cores were collected. Ebullition samples were taken in water depths varying from 30 to 80 cm according to spatial changes in the water level. To collect the samples we modified the method described by Martens et al. (1992). The gas was collected in an inverted funnel (20 cm diameter) and transferred into a 6 ml gas tight syringe. The gas samples (2 ml) were then transferred into 12-ml glass vials containing N_2_ previously sealed with butyl rubber stopper. Nine samples were sent for carbon isotopic analysis of methane and carbon dioxide to the Max-Planck Institute for terrestrial Microbiology, Marburg (Germany).

### Chemical and isotopic analyses

CH_4_ was analyzed by gas chromatography (GC) using a flame ionization detector (Shimadzu, Kyoto, Japan). CO_2_ was analyzed after conversion to CH_4_ with a methanizer (Ni-catalyst at 350°C, Chrompack, Middelburg, Netherlands). Isotope measurements of ^13^C/^12^C in gas samples were performed on a gas chromatograph combustion isotope ratio mass spectrometer (GC-C–IRMS) system (Thermo Fisher Scientific, Bremen, Germany). The principle operation was described by Brand (1996). The gaseous compounds were first separated in a Hewlett Packard 6890 GC using a Pora Plot Q column (27.5 m length, 0.32 mm internal diameter, and 10 μm film thickness; Chromopack Frankfurt, Germany) at 30°C and He (99.996% purity; 2.6 ml/min) as carrier gas. The sample was run through the Finnigan Standard GC Combustion Interface III and the isotope ratio of ^13^C/^12^C was analyzed in the IRMS (Finnigan MAT Deltaplus). The reference gas was CO_2_ (99.998% purity) (Air Liquide, Düsseldorf, Germany), calibrated with the working standard methylstearate (Merck). The latter was intercalibrated at the Max Planck Institute for Biogeochemistry, Jena, Germany (courtesy of W. A. Brand) against the NBS-22 and USGS-24 standards and reported in the delta notation vs. Vienna Pee Dee Belemnite.

(3) $$\delta^{\text{13}}{\text{C = 10}}^{\text{3}}({\text{R}}_{\text{sample}}{\text{/R}}_{\text{standard}}-\text{1})$$

with R = ^13^C/^12^C of sample and standard, respectively.

Isotopic analysis and quantification of acetate were performed on a high pressure liquid chromatography (HPLC) system (Spectra System P1000, Thermo Fisher Scientific, San Jose, CA, USA; Mistral, Spark, Emmen, the Netherlands) equipped with an ion-exclusion column (Aminex HPX-87-H, BioRad, München, Germany) and coupled to Finnigan LC IsoLink (Thermo Fisher Scientific, Bremen, Germany) as described (Krummen et al., 2004). Isotope ratios were detected on an IRMS (Finnigan MAT Deltaplus Advantage).

The δ^13^C in the organic matter was analyzed at the University of Göttigen (Germany) using an elemental analyzer (Fisons EA 1108) coupled to a mass spectrometer. The C, N, and H content of the sediments were quantified on a CHNS-element analyzer by the Analytical Chemical Laboratory of the University of Marburg.

### Calculations

The carbon isotopic signature was given in the delta notation relative to the Vienna Pee Dee Belemnite (V-PDB) standard. The fractionation factor α for a reaction A → B are defined after (Hayes, 1993):

(4) $$\alpha_{\text{A},\text{ B}}\text{=}(\delta^{\text{13}}{\text{C}}_{\text{A}}{\text{+10}}^{\text{3}})\text{/}(\delta^{\text{13}}{\text{C}}_{\text{B}}{\text{+10}}^{\text{3}})$$

Isotopic calculations of fractionation factors and estimation of the approximate partition of hydrogenotrophic methanogenesis of the total methanogenesis were calculated according to Conrad (2005):

The apparent fractionation factor (α_app_) for conversion of CO_2_ to CH_4_ is given by:

(5) $$\alpha_{\text{app}}\text{=}(\delta {\text{CO}}_{\text{2}}{\text{+10}}^{\text{3}})\text{/}(\delta {\text{CH}}_{\text{4}}{\text{+10}}^{\text{3}})$$

where δCO_2_ and δCH_4_ are directly measured isotopic signatures of the carbon in CO_2_ and CH_4_, respectively.

Fractionation factor for hydrogenotrophic methanogenesis (α_mc_) is given by:

(6) $$\alpha_{\text{mc}}\text{=}(\delta {\text{CO}}_{\text{2}}{\text{+10}}^{\text{3}})\text{/}(\delta_{\text{mc}}{\text{+10}}^{\text{3}})$$

where δ_mc_ is carbon isotopic signature of methane solely produced from carbon dioxide (directly measured from assays inhibited by methyl fluoride). Partition of hydrogenotrophic methanogenesis is calculated by the following mass balance Equation (7):

(7) $$f_{\text{mc}}=(\delta_{\text{CH4}}-\delta_{\text{ma}})\text{/}(\delta_{\text{mc}}-\delta_{\text{ma}})$$

where f_mc_ is the partition of hydrogenotrophic methanogenesis and δ_ma_ is carbon isotopic signature of methane solely produced from acetate. It is calculated from the following equation:

(8) $$\delta_{\text{ma}}\text{ = }(\text{1/}\alpha_{\text{ma}})(\delta_{\text{ac}}{\text{+10}}^{\text{3}}-\alpha_{\text{ma}}{}^{*}{\text{10}}^{\text{3}})$$

where α_ma_ is fractionation factors for acetoclastic methanogenesis and δ_ac_ is the measured isotopic signal of acetate. Several published α_ma_ have been used to estimate the contribution of hydrogenotrophic methanogenesis e.g., (Gelwicks et al., 1994; Penning et al., 2006; Goevert and Conrad, 2009).

### Molecular analyses

DNA was extracted from the fresh sediment before the start of the incubation and at the end of the incubations (with and without methyl-fluoride) using the PowerSoil DNA Isolation Kit (MO BIO, USA), according to the manufacturer's instructions. The extracted DNA was used to characterize the *mcrA* gene by T-RFLP (Terminal-restriction length polymorphism) according to Chin et al. (Liu et al., 1997; Chin et al., 1999) using the primers *mcrA* f (TAY GAY CAR ATH TGG YT) and *mcrA* r (ACR TTC ATN GCR TAR TT) published by Springer et al. (1995) with a FAM (6-carboxyfluorescein)-label at the forward primer. The *mcrA* gene amplicons were digested with Sau96I (Fermentas), and the products were size-separated in an ABI 3130 DNA sequencer (Applied Biosystems, Darmstadt, Germany). For downstream analysis only fragments between 80 and 520 bp have been considered to avoid analysis of false signals originated from primer residuals, primer dimmers, and undigested PCR product. The normalization and standardization of the T-RFLP profiles was done according the method from Dunbar et al. (2001). The relative abundance was calculated using the ratio between the height of the fluorescence signal and the total height of all signals in one sample. To assign the resulting fragments we used a clone library which was constructed in our lab in a framework to characterize the methanogenic community at different locations and depth of River Sitka (Figure S9). The dominant peaks well-reflect published literature values of other water logged systems (Lueders et al., 2001; Ramakrishnan et al., 2001; Chin et al., 2004; Kemnitz et al., 2004; Conrad et al., 2008).

## Results

**Figure 1:**
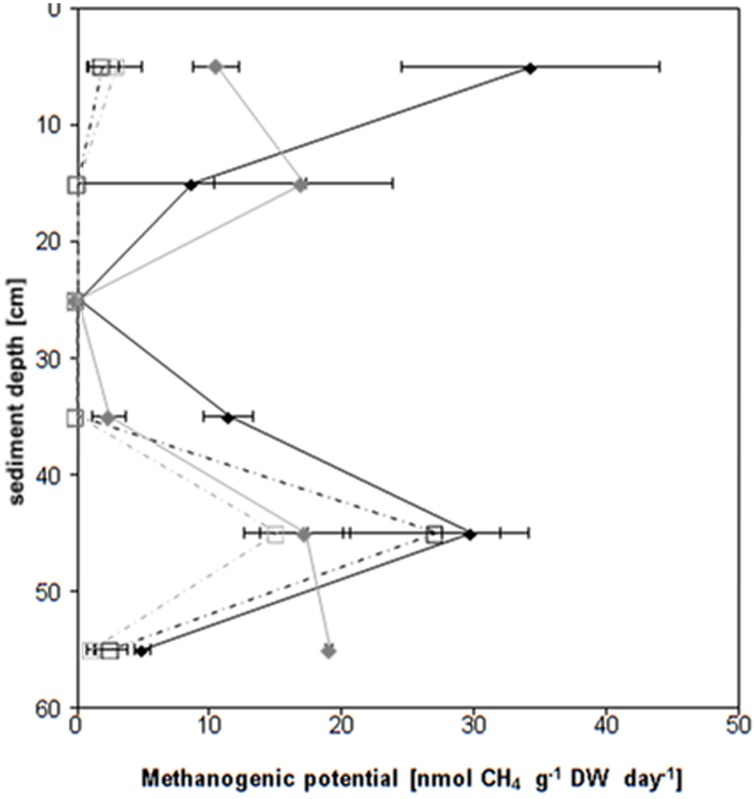
**Vertical profile (60 cm depth sampled in 10 cm slices) of the methanogenic potential of two sediment cores**. Core I uninhibited control N_2_♦, core I methyl fluoride (N_2_ + 3% CH_3_F)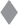, core II uninhibited control N_2_□, core II methyl fluoride (N_2_ + 3% CH_3_F)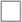. The methanogenic potential (in nmol per g dry weight (DW) per day) has been calculated using the slope of the methane concentration over the last 10–11 day of the incubation (compare Figure S1). The values of the individual layers (e.g., 0–10 cm) are given as average (e.g., 5 cm) The rates are given ± standard deviation (*n* = 3-5).

All samples of core I and almost all samples of core II (except the 20–30 and 30–40 cm depth and the 10–20 cm depth under inhibited conditions) released methane and all samples released carbon dioxide under the chosen incubation conditions (Figure 1, Figures S1, S2). Both cores showed the same vertical pattern of methane emission rates (Figure 1, Figure S1): The highest average methane production rates (up to 34 ± 11 nmol CH_4_ g^−1^ DW day^−1^) were found in the top 10 cm and in the 40–50 cm depth layer. The 10–40 cm depth layers as well as the 50–60 cm depth layer proved low methane production rates (below 9 ± 9 nmol CH_4_ g^−1^ DW day^−1^) for the first core and negligible if any methane production for the second core. Roughly threefold more methane was released under uninhibited conditions in the top 10 cm; the 40–50 cm peak was doubled in the absence of methyl fluoride. In the presence of methyl fluoride methane production rates followed the same pattern, again showing the highest values in the 40–50 cm depth layer of both cores.

The concentrations of free carbon dioxide in the headspace increased in all sediment layers under all tested treatments in both cores (Figure S2). During the methanogenic lag phase carbon dioxide concentrations of both cores increased faster and later on the increase was slowing down up to the end of incubation. The upper 10 cm of both cores showed the highest concentrations. Generally methyl fluoride amendment did not systematically affect the carbon dioxide concentrations.

**Figure 2:**
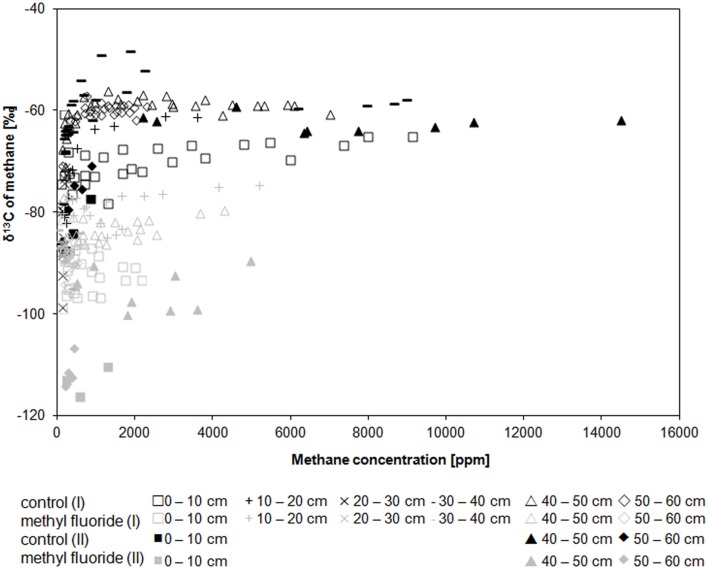
**Course of the δ^13^C of CH_4_ along CH_4_ concentrations in both inhibited and uninhibited control assays for core (I) open symbols and core (II) filled symbols**. Uninhibited control incubations (N_2_) in black, methyl fluoride inhibited samples (N_2_ + 3% CH_3_F) in gray. Details are likewise plotted in Figures S1, S3.

In both cores the δ^13^C of methane for uninhibited controls was in the range of −98.6 to −48.2‰ and for inhibited incubations in the range of −116.3 to −74.5‰ (Figure 2, Figure S3). The δ^13^C of methane was not affected by the sampling depth. The *in-situ* δ^13^C of methane (−59.0 ± 1.2 ‰, *n* = 9) was very close to the methane measured in the maximum methanogenic depth layers for uninhibited control assays (−59 to −62‰). The δ^13^C of carbon dioxide was irrespective of the treatment during the incubations in the range of −18.8 to −36.0‰ for all depth layers and both cores (Figure S4). The initially light CO_2_ (−18.8 to −25.3‰) usually became heavier during the incubation; only the samples showing high methane production rates had lighter CO_2_ in the end (Figure S4). The *in-situ* δ^13^C carbon dioxide was slightly heavier (−16.3 ± 1.2‰, *n* = 9).

**Figure 3:**
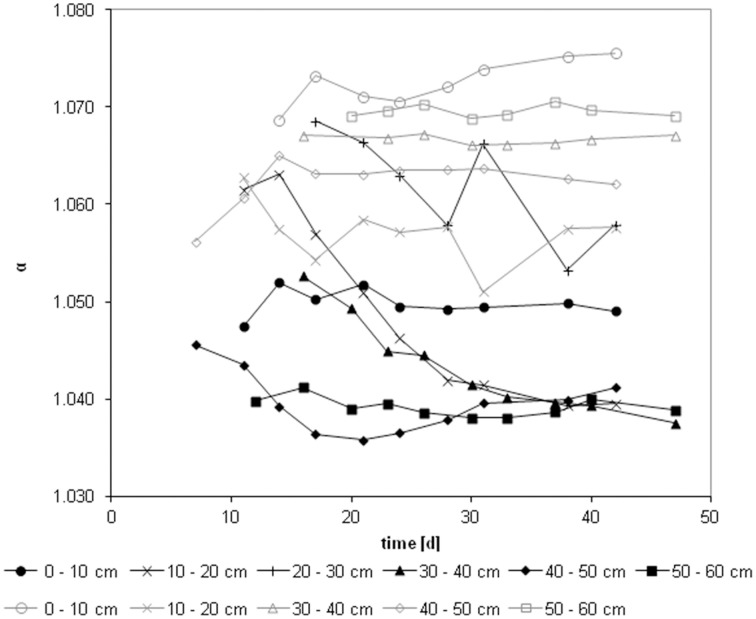
**Course of fractionation factors during incubations: apparent fractionation factor(α_app_) for the core I**. Uninhibited incubations in black, methyl fluoride inhibited samples in gray.

We calculated the apparent fractionation (α_app_) for uninhibited control and inhibited samples using Equations (5) and (6), respectively, (Figure 3). While the apparent fractionation of core I for the uninhibited samples was on average 1.046 ± 0.009 (*n* = 54) ranging from 1.039 (50–60 cm) to 1.062 (20–30 cm), the inhibited samples were approximately 20‰ more depleted in ^13^C: 1.065 ± 0.006 (*n* = 43) ranging from 1.057 (10–20 cm) to 1.073 (0–10 cm). Only three depth layers (0–10, 40–50, and 50–60 cm) of core II could be fully evaluated using prolonged incubation times (30–80 days). The apparent fractionation of the uninhibited samples ranged from 1.039 to 1.065. The inhibited samples again were approximately 29‰ more depleted in ^13^C and ranged from 1.069 to 1.088. It is worth noting that the two depth layers with the highest methane production potentials showed distinct apparent fractionations: in the top layer the average apparent fractionation was 1.073 in the inhibited and 1.050 in the uninhibited samples; in the 40–50 cm depth layer the apparent fractionation was 1.062 and 1.040, respectively. The average apparent fractionation factor for the *in situ* samples was 1.045 ± 0.002 (*n* = 9).

Carbon contents in incubated sediments are listed in Table 2. They showed no vertical pattern but differed in the two sediment cores. The average carbon isotope values of organic matter was −26.3‰ (± 0.1‰, *n* = 12). The acetate concentrations at the end of the incubations stayed at a relatively low level (<0.02 mM uninhibited; up to 1.5 mM under CH_3_F) again showing a peak in the top 10 cm and for the 40–50 cm depth layer. The δ^13^C values of acetate were in the range of −50.7 to −31‰ and −30.8 to −27.5‰ for inhibited and uninhibited incubation assays respectively, (Table 2). For all sediment samples, the δ^13^C of produced acetate was lower than the δ^13^C of organic matter. Other parameters (e.g., H and N content) are listed in Table S1.

**Table 2:** **Depth profiles of two sediment cores**.

**Depth [cm]**	**C_soil_** [%]	**δ^13^C_soil_ [‰]**	**Uninhibited**		**methylfluoride**	
			**acetate [mM]**	**δ^13^C_acetate_ [‰]**	**acetate [mM]**	**δ^13^C_acetate_ [‰]**
**CORE I**.						
0−10	0.6	−26.2	n.d.	n.d.	1.53 ± 0.6	−42.0 ± 1.2
10−20	2.3	−25.9	n.d.	n.d.	0.47 ± 0.17	−47.8 ± 7.9
20−30	0.9	−25.8	n.d.	n.d.	n.d.	n.d.
30−40	2.9	−26.8	0.02 ± 0.00	−27.4 ± 1.4	0.58 ± 0.16	−34.9 ± 1.9
40−50	2.3	−26.8	0.03 ± 0.00	−27.8 ± 0.2	0.79 ± 0.07	−34.3 ± 1.0
50−60	1	−26.3	0.02 ± 0.00	−29.6 ± 0.4	0.08 ± 0.06	−31.0 ± 1.2
**CORE II**.						
0−10	0.5	−26.4	n.d.	n.d.	0.31 ± 0.41	−50.7 ± 1.0
10−20	0.7	−26.3	n.d.	n.d.	n.d.	n.d.
20−30	0.9	−26.3	n.d.	n.d.	n.d.	n.d.
30−40	0.7	−26	n.d.	n.d.	n.d.	n.d.
40−50	6.8	−26.7	0.02 ± 0.00	−27.5 ± 1.0	1.16 ± 0.27	−40.7 ± 4.9
50−60	2.4	−26.2	n.d.	n.d.	0.02 ± 0.01	−38.6 ± 1.3

*Soil carbon content and delta ^13^C values of the original sediments are given together with the acetate concentrations and isotopic signals of uninhibited and inhibited incubation (CH_3_F) experiments at the end of the incubation. n.d. not detected. Values are given ± standard deviation n = 3*.

**Figure 4:**
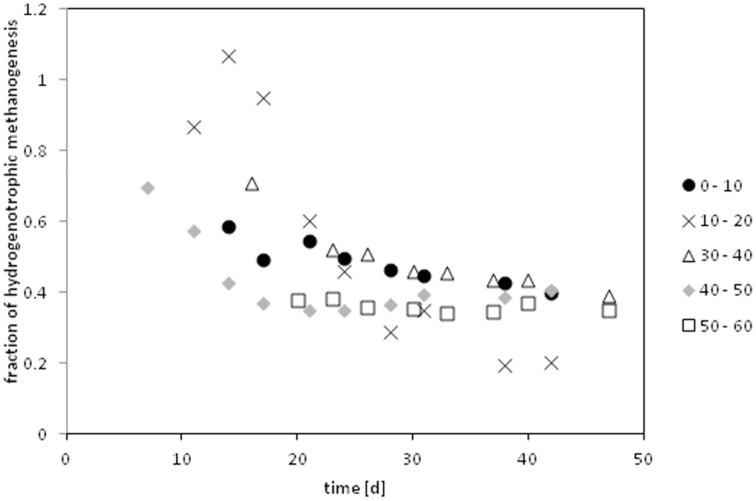
**Relative contribution of hydrogenotrophic methanogenensis to the released methane of the depth profile of core I**. Calculated assuming a fractionation factor of α_app_ = 1.009 for acetoclastically produced methane. (Compare Figures S5, S6). The 20–30 cm depth did not release methane under inhibited conditions; hence the contribution of hydrogenotrophic methanogens could not be calculated for that sample.

The contribution of hydrogenotrophic methanogenesis (f_mc_) was calculated by Equation (7) incorporating measured δ^13^C of methane (δ_CH4_), methane produced purely from hydrogenotrophic methanogenesis (δ_mc_) and an estimate for the methane produced from acetate (δ_ma_) based on measured ^13^C acetate and fractionation factors of acetoclastic methanogenesis presented in literature. The time courses of f_mc_ in the core (I) calculated with α_ma_ = 1.009 (Goevert and Conrad, 2009) is shown in Figure 4. In the beginning almost all methane was produced from hydrogen; later the contribution of hydrogenotrophic methanogenesis dropped to about 40%. In core II only three depth layers could be evaluated during the second half of the incubation period. These samples showed a contribution of 26–45% of hydrogenotrophic methanogenesis to the released methane.

**Figure 5:**
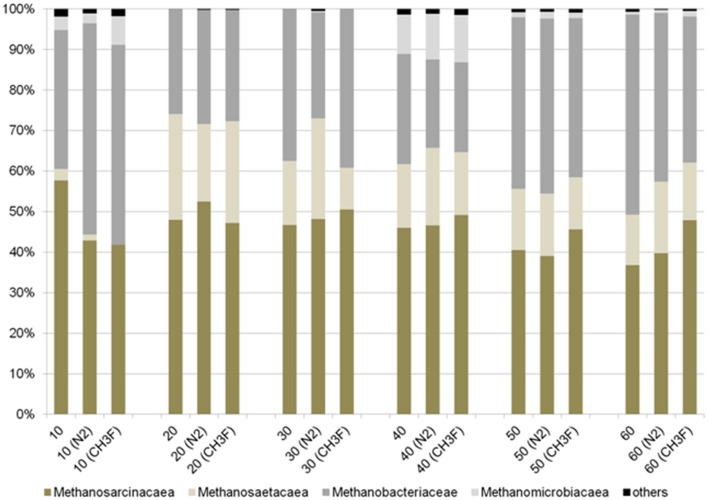
**Relative abundance of important methanogenic groups as determined by T-RFLP for core I (different depth layers up to 60 cm depth)**. Results are given for samples before the incubation as well as at the end of incubations under N_2_ (control incubations) or N_2_ + 2% CH_3_F (inhibition of acetoclastic methanogenesis). Details on T-RFLP results are given in Figure S8.

The molecular analysis of the methanogenic marker-gene (*mcrA*) revealed a significant different methanogenic community for the top layer in contrast to deeper layers (Figure 5). The community profile (T-RFLP of *mcrA*) resolves in up to 11 fragments (Figure S8). The microbial community was not affected by the incubation under N_2_ or N_2_ + CH_3_F. In all depth layers and under all incubation conditions *Methanosarcinacea* were the dominant group (22–52%) followed by *Methanobacteriacea* (24–56%); *Methanomicrobiales* were only detectable in the two active layers (up to 12%). *Methanosaetacea* were almost absent in the top layer (below 3%) and reached a higher relative abundance in deeper layers (10–25%). The samples of core II have not been analyzed by T-RFLP. However, a core sampled in 2014 at the same location did confirm the overall pattern of the T-RFLP but showed a more gradual change of the community over the depth profile.

## Discussion

### Methane production potentials in river sediments

Estimations of the methane production potentials of river sediments have so far only been made for mixed top sediments: e.g., White Oak River sediment incubations at 25°C had methane production potentials of approximately 250 nmol gDW^−1^ d^−1^ (originally given as 8 μM hr^−1^) (Avery and Martens, 1999). Incubations of fresh top sediment layers or river Sitka sampled in spring 2014 and incubated under similar conditions as reported in this study resulted in more than tenfold larger methane production potentials of 469 nmol gDW^−1^ d^−1^ (Bednarik unpublished) compared to 34 nmol CH_4_ gDW^−1^ d^−1^ reported for the top 10 cm in this study. While top sediments of the Elbe River had maximum potential methane production rates of 552 nmol gDW^−1^ d^−1^ (Matoušů in preparation). This would suggest that the methane production potential of river sediments reaches methane production potentials up to 552 nmol gDW^−1^ d^−1^. For comparison lake sediments have a methane production potential of e.g., 9–3380 nmol gDW^−1^ d^−1^ (Conrad et al., 2010, 2011) while rice field soils show methane production rates of 3360–7920 nmol gDW^−1^ d^−1^ (Conrad et al., 2009a, 2012).

In order to better understand the methanogenic potential of river sediments we incubated several depth layers of two sediment cores under anoxic conditions in the laboratory. While published data of *in situ* measurement of methane concentrations in river sediments pointed to diverse vertical profiles, reaching several hundred μM (Table 3) it is as well-possible that the *in situ* concentrations are independent from the underlying methane production potential.

**Table 3:** **Methane concentration in depth profiles of river sediments**.

**River**	**Sample description**	**Depth (cm)**	**CH_4_ concentration (μM)**	**References**
Hudson	Station 118 Aug. 23, 1991	50	0.105	De Angelis and Scranton, 1993
		1000	0.99	De Angelis and Scranton, 1993
		2000	0.108	De Angelis and Scranton, 1993
Severnaya Dvina River	Station 20	0-5	0.5	Gar'Kusha et al., 2010
(White Sea)		5–10	3	Gar'Kusha et al., 2010
Allequash Creek	Lower site	20	430	Schindler and Krabbenhoft, 1998
(Wisconsin, USA)		60	410	Schindler and Krabbenhoft, 1998
		180	400	Schindler and Krabbenhoft, 1998
	Upper site	top	2	Schindler and Krabbenhoft, 1998
Jiulong River Estuarine		90	6	Chen and Yin, 2013
		100–140	2–3	Chen and Yin, 2013
		150	6	Chen and Yin, 2013
Sitka	Location IV	10	20	Rulík et al., 2013
		20	175	Rulík et al., 2013
		30	300	Rulík et al., 2013
		40	175	Rulík et al., 2013
		50	260	Rulík et al., 2013

Indeed we find two distinctive peaks of methane production in the present study (up to 34 nmol gDW^−1^ d^−1^) which correlated with higher CO_2_ production in these layers and acetate accumulation in the inhibited samples. These peaks are present in both cores which have been separately analyzed. The earlier reported preliminary methane production potentials for River Sitka under substrate addition (ca. 8 μM acetate) were much lower (below 6 nmol gDW^−1^ d^−1^) and only based on two time points and a very short incubation time (72 h) (Rulík et al., 2013). Since we could show that roughly 40% of methane is produced hydrogenotrophically, these short time incubations under substrate addition may not reflect the natural conditions. However, already these incubations showed two distinct peaks for the top sediment and 40–50 cm depth. In this respect it is worth to note that the lag phase of our incubation experiments lasted for about 15–35 days (Figure S1), which is most probable due to the presence of other electron acceptors which have to be depleted before methanogenesis starts.

The methane production potential of the top layer is paralleled by high oxygen saturation (>80%) (Rulík et al., 2013), low *in situ* methane concentrations (Table 3) and high activities of methanotrophic bacteria [Figure S7 and (Rulík et al., 2013)]. The second peak goes along with lower oxygen saturation (17.5%) (Rulík et al., 2013), intermediate *in situ* methane concentration, and reduced methanogenic activity. However, it is presently not clear why the intermediate zone (10–30cm) shows almost no methanogenic potential.

### Methanogenic pathways in river sediments

Our result shows that carbon isotopic values of methane measured both *in situ* (−59‰) and in different incubations of depth layers (−68 to −59‰) were in the broad range of δ^13^C of methane measured in other studies in rivers e.g., *in situ* measurements from the Amazonian rivers ranged from −75 to −53‰ (Moura et al., 2008) but slightly heavier than methane collected from interstitial water at 40–50 cm depth in Sitka (−72 to −68‰) (Rulík et al., 2013).

Assuming complete inhibition of acetoclastic methanogenesis in the presence of methyl fluoride (CH_3_F) the isotopic signal of the methane can be completely attributed to hydrogenotrophically produced CH_4_ (δ_mc_). The range for the apparent fractionation reported in our study (α_app_ = 1.04 to 1.06) have quite commonly been observed in e.g., rice field soils (Sugimoto and Wada, 1993; Chidthaisong et al., 2002; Penning and Conrad, 2007; Conrad et al., 2009b).

The fractionation factor (α_ma_) during conversion of total acetate to methane in *Methanosarcina acetivorans* and *M. barkeri* ranges from α_ma_ of 1.012–1.027 (Gelwicks et al., 1994; Conrad, 2009; Goevert and Conrad, 2009), whereas isotope fractionation in *Methanosaeta* spp. is weaker, i.e., α_ma_ of 1.007–1.009 (Valentine et al., 2004; Penning et al., 2006). From an earlier study, it was found that both acetoclastic genera *Methanosarcina* spp. and *Methanosaeta* spp. occur in Sitka sediments (Buriankova et al., 2013). Therefore, we calculated the contribution of hydrogenotrophic methanogenesis with all published fractionation factors ranging up to α_ma_ = 1.027 (Figure S5). However, α_ma_ = 1.009 is maybe most reasonable because fractionation factors of acetoclastic methanogenesis under environmental settings are approximately 5–10‰ less negative than in pure culture, which is probably due to limitation by acetate (Penning and Conrad, 2007; Conrad, 2009; Goevert and Conrad, 2009). We used the isotopic value of the total acetate for our calculations, which may be incorrect since we find a high contribution of acetogenesis to the acetate pool (compare Discussion below). If we use the isotopic signal of the soil organic carbon as a proxy for the acetate values we get almost the same results (Figure S6 and accompanying discussion). When carbohydrates are methanogenically degraded *f*_mc_ is expected to be 33%, which is commonly observed in e.g., rice field soils (Conrad, 1999). Other environments like e.g., lake sediments can have much larger contributions of hydrogenotrophically produced methane (Conrad, 1999). Estimates of *f_mc_* for White Oak River sediments were reported to be 37–39% (Avery and Martens, 1999) which is in good agreement with our own results (40%). Comparing the different layers of our depth profile it was found that the upper maximum (0–10 cm) produce slightly more hydrogenotrophic methane 42–51% than the 40–50 cm layer 36–46%.

### Isotope fractionation during acetate production

While the major sink of acetate in methanogenic environments is methane two dominant mechanisms are known to replenish the acetate pool: Acetate is produced either by fermentation of organic matter or by reduction of CO_2_ with H_2_ via the acetyl-CoA pathway (acetogenesis) (Drake et al., 2006). Hence the *in-situ* δ^13^C value of acetate is influenced by all three reactions (Heuer et al., 2010; Conrad et al., 2014). Acetoclastic methanogenesis has a moderate fractionation around α = 1.01 (see Discussion above), fermentation has only a very weak preference for either carbon isotope [α < 1.009 (Blair et al., 1985; Penning and Conrad, 2006)], a stronger preference for light carbon has been determined for the acetyl-CoA pathway [α = 1.06 (Gelwicks et al., 1989; Blaser et al., 2013)]. In principle syntrophic acetate oxidation coupled to hydrogenotrophic methanogenesis is an alternative route to deplete acetate (Zinder and Koch, 1984; Conrad and Klose, 2011; Rui et al., 2011; Dolfing, 2014).

As a result of all three reactions the acetate signatures in environmental samples are usually in the range of the soil organic carbon (± 10‰) e.g., (Conrad et al., 2011, 2014). The presence of methyl fluoride blocks the only acetate depleting reaction (in our experimental set up) and hence results in an accumulation of acetate. In most studies this acetate however does no significantly differ from the acetate signature of the uninhibited control incubations under N_2_ e.g., (Heuer et al., 2010; Conrad et al., 2011). In our sample the acetate signatures of the uninhibited samples are similar to the ^13^C values of soil organic carbon, while inhibited samples are always depleted in ^13^C relative to the soil organic carbon (−5 to −24‰; compare Table 1). This may point to a relative high contribution of the strong fractionating acetyl-CoA pathway to the acetate signature under these conditions.

If we assume complete inhibition of acetoclastic methanogenesis in these samples and no fractionation during fermentation, the contribution of the acetyl-CoA pathway can be calculated to be 8–41% (Table S2). If a stronger fractionation during fermentation (α = 1.01) is assumed the contribution is between 0 and 29%. In comparison we calculated a lower contribution of acetogenic bacteria for data published by Conrad et al. (2011) on anoxic lake sediments: 0–19% (no fractionation scenario) or 0–3% (α = 1.01).

Under methyl fluoride inhibition the acetyl-CoA pathway competes with hydrogenotrophic methanogenesis for the substrates hydrogen and carbon dioxide which are either reduced to acetate or to methane. Since methanogenesis is energetically more favorable than acetogenesis (131 vs. 95 kJ mol^−1^) it outcompetes acetogenesis in many environments (Kotsyurbenko et al., 2001). Acetogenesis can become dominating under elevated hydrogen partial pressures: e.g., Heuer et al. reported strongly depleted acetate (δ_acetate_ = −48.8‰ for lake sediments incubated under elevated hydrogen partial pressure (Heuer et al., 2010). Likewise low temperatures favor the prevalence of acetogens over hydrogenotrophic methanogens (Kotsyurbenko et al., 2001). Oxygen is a third factor in favor of acetogenic bacteria which are better adopted to aerated environments than methanogens (Kuesel and Drake, 1995).

Our data suggest that acetogenic bacteria contribute up to 40% of the produced acetate in river sediments (under CH_3_F inhibition) and that they can effectively compete with hydrogenotrophic methanogens. Therefore, acetogens may play an important yet not well-characterized role in river sediment ecology.

### Methanogenic community profile

The methanogenic community based on T-RFLP of *mcrA* has so far primarily been described for rice field soils (Lueders et al., 2001; Ramakrishnan et al., 2001; Chin et al., 2004; Kemnitz et al., 2004; Conrad et al., 2008). Most of the fragments we found in the clone library of river systems were identical with previously published T-RF's. The only exception was the 473 bp fragment, which is distinct from the 470 bp fragment of *Methanobacteria* (Lueders et al., 2001; Chin et al., 2004) and could be assigned to the order of *Methanomicobiales* using cloning and sequencing (Figure S9). This fragment was only present in the two layers showing high methanogenic potentials. The absence of *Methanosaetacea* in the top layer is plausible since they are commonly found in permanent anoxic systems like fresh water sediments (Banning et al., 2005; Chan et al., 2005; Youngblut et al., 2014) but only dominate in rice paddies when acetate is scarce (Lueders et al., 2001; Ramakrishnan et al., 2001; Chin et al., 2004; Kemnitz et al., 2004; Conrad et al., 2008). This has been attributed to a reduced stress tolerance of these strains e.g., lower oxygen tolerance (Erkel et al., 2006; Yuan et al., 2011). Molecular data based on the *mcrA* gene suggest that the methanogenic community is stable over the depth (ca. 10^7^ *mcrA* copies g^−1^ DW, Chaudhary et al., in preparation). Likewise the pathway usage (compare Discussion above) is only mildly affected by the sediment depth. It is therefore most plausible that the differences in the methane production potential are caused by the activity of different methanogenic archaea and may as well be influenced by substrate availability. Indeed the 40–50 cm depth peak has the highest organic carbon content in core II (compare Table 2).

Our study revealed no difference in the T-RFLP profiles before and after incubations suggesting that the methanogenic community was rather stable over the approximately 2 month incubation period. Similar results have been found for rice field soil incubations (Yuan et al., 2011; Ma et al., 2012) and river sediment (Beckmann and Manefield, 2014). It can therefore be assumed that the differences in the methanogenic potential are regulated on the RNA or activity level of *mcrA* rather than caused by growth of the methanogenic archaea. This would also explain why the second methanogenic peak in the potential measurements (40–50 cm) could not be anticipated by the molecular data alone. The presence of methyl fluoride did not impact the T-RFLP profiles. This is in agreement with Daebeler et al. which showed that the presence of methyl-fluoride impacts the methanogenic activity rather than changing the community composition of methanogenic archaea (Daebeler et al., 2013).

## Conclusions

Our experiments show that methane is produced in anoxic incubations of river sediment cores. Methane production is vertically organized showing two distinct maxima in the top layers and in 40–50 cm depth. The magnitude of the calculated methane production rates in rivers covers a broad range but is on average lower than the reported potential of other water logged systems (lakes, rice paddies). Likewise, the pathway usage (contribution of hydrogenotrophic methanogenesis) is comparable to previously studied fresh water systems. Under methyl fluoride inhibition the ^13^C value of acetate is unusually light pointing to a high contribution of acetogenic bacteria. The methanogenic community composition was different in the top sediment while the lower segments share similar methanogenic fingerprints.

### Conflict of interest statement

The authors declare that the research was conducted in the absence of any commercial or financial relationships that could be construed as a potential conflict of interest.
