# Supplementary material for: Methane production potentials, pathways, and communities of methanogens in vertical sediment profiles of river Sitka
Source: Front Microbiol. 2015 May 21;6:506. doi: 10.3389/fmicb.2015.00506 (PMC4440369; doi:10.3389/fmicb.2015.00506)
Supplement: Supplementary file 1 [file DataSheet1.DOCX]

**Methane production potentials, pathways, and communities of methanogens in vertical sediment profiles of river Sitka**

Supplementary

Vaclav Mach^1,3^, Martin Blaser^2,3^, Peter Claus^2^, Prem Prashant Chaudhary^1^, Martin Rulík^1^

*1) Department of Ecology and Environmental Science, Faculty of Science, Palacky University, Olomouc, Czech Republic*

*2) Department of Biogeochemistry, Max Planck Institute for Terrestrial Microbiology, Marburg, Germany*

*3) These authors contributed equally to this work*

*Corresponding author:

Martin Blaser

Max Planck Institute for Terrestrial Microbiology

Karl-von-Frisch-Str. 10

35043 Marburg, Gemany

Tel: +49-6421-178 870

Fax: +49-6421-178 999

Email: blaserm@mpi-marburg.mpg.de

Table S1 Nitrogen and hydrogen content ± SE (n=2 each) for the Sitka sediment at different depth for two sediment cores (I and II). The nitrogen isotope values are given as well. Isotopic data has only been measured in one sample per depth layer.

|  | H [%] | | N [%] | | δ^15^N [‰] | |
| --- | --- | --- | --- | --- | --- | --- |
| Depth | I. | II. | I. | II. | I. | II. |
| 0 - 10 | 0.30 ± 0.02 | 0.29 ± 0.005 | 0.03 ± 0.00 | 0.25 ± 0.01 | 6.01 | 6.28 |
| 10 - 20 | 0.47 ± 0.01 | 0.30 ± 0.015 | 0.10 ± 0.00 | 0.40 ± 0.00 | 5.07 | 5.07 |
| 20 - 30 | 0.32 ± 0.01 | 0.35 ± 0.015 | 0.05 ± 0.01 | 0.55 ± 0.01 | 5.34 | 5.66 |
| 30 - 40 | 0.62 ± 0.02 | 0.33 ± 0.005 | 0.17 ± 0.00 | 0.40 ± 0.00 | 5.33 | 5.55 |
| 40 - 50 | 0.57 ± 0.02 | 1.18 ± 0.01 | 0.16 ± 0.01 | 0.42 ± 0.01 | 5.33 | 5.61 |
| 50 - 60 | 0.34 ± 0.01 | 0.55 ± 0.01 | 0.06 ± 0.01 | 0.13 ± 0.01 | 5.73 | 5.49 |

Table S2 contribution of acetogenic versus fermentative acetate under methylfluorid inhibition.

| Core I. |  |  |  | contribution of acetogens | |
| --- | --- | --- | --- | --- | --- |
| depth [cm] | δ^13^C_acetate_ [‰] | δ^13^C_soil_ [‰] |  | Szenario I [%] | Szenario II [%] |
|  |  |  |  |  |  |
| 0 - 10 | -42 | -26.2 |  | 12 | 26 |
| 10 - 20 | -47.8 | -25.9 |  | 24 | 37 |
| 20 - 30 | n.d. | -25.8 |  | n.d. | n.d. |
| 30 - 40 | -34.9 | -26.8 |  | -4 | 14 |
| 40 - 50 | -34.3 | -26.8 |  | -5 | 13 |
| 50 - 60 | -31 | -26.3 |  | -11 | 8 |
|  |  |  |  |  |  |
| Core II. |  |  |  | contribution of acetogens | |
| depth [cm] | δ^13^C_acetate_ [‰] | δ^13^C_soil_ [‰] |  | Szenario I [%] | Szenario II [%] |
|  |  |  |  |  |  |
| 0 - 10 | -50.7 | -26.4 |  | 29 | 41 |
| 10 - 20 | n.d. | -26.3 |  | n.d. | n.d. |
| 20 - 30 | n.d. | -26.3 |  | n.d. | n.d. |
| 30 - 40 | n.d. | -26 |  | n.d. | n.d. |
| 40 - 50 | -40.7 | -26.7 |  | 8 | 23 |
| 50 - 60 | -38.6 | -26.2 |  | 5 | 21 |
|  |  |  |  |  |  |
|  |  |  |  |  |  |
|  |  |  |  | contribution of acetogens | |
| lake^1^ | δ^13^C_acetate_ [‰] | δ^13^C_soil_ [‰] |  | Szenario I [%] | Szenario II [%] |
| 1 | -14.5 | -25.2 |  | -41 | -18 |
| 2 | -16 | -27.1 |  | -42 | -19 |
| 3 | -25 | -29.3 |  | -29 | -7 |
| 4 | -21.6 | -28 |  | -33 | -11 |
| 5 | -34.6 | -27.9 |  | -7 | 11 |
| 6 | -34.3 | -27.6 |  | -7 | 11 |
| 7 | -22.6 | -26.9 |  | -29 | -7 |
| 8 | -28.5 | -29.2 |  | -21 | -1 |
| 9 | -20.8 | -30.1 |  | -39 | -16 |
| 10 | -24.5 | -31.3 |  | -34 | -11 |
| 11 | -23.7 | -29.5 |  | -32 | -10 |
| 12 | -19.1 | -28.6 |  | -39 | -16 |
| 13 | -28.5 | -29.1 |  | -21 | -1 |
| 14 | -33.7 | -29.5 |  | -12 | 7 |
| 15 | -43.8 | -32.4 |  | 3 | 19 |
| 16 | -36.5 | -32.8 |  | -13 | 6 |

1) Data and lake nomenclature taken from: ([Conrad et al., 2011](#_ENREF_4))

Scenario I has been calculated using a fractionation of ε_ac_=-60‰ (α = 1.060) for acetogens ([Gelwicks et al., 1989](#_ENREF_5);[Blaser et al., 2013](#_ENREF_2)) and ε_ferm_=-10‰ (α = 1.010) for fermentation ([Blair et al., 1985](#_ENREF_1);[Penning and Conrad, 2006](#_ENREF_7)) using: (δ^13^C_acetate_ – δ^13^C_soil_ - ε_ferm_) / ε_ac_; Scenario II has been calculated using a fractionation of α = 1.060 for acetogens and no fractionation during fermentation using (δ^13^C_acetate_ – δ^13^C_soil_) / ε_ac_. In both scenarios the soil organic carbon was defined as substrate; the isotopic signal of acetate under methylfluoride inhibition was defined as product. Negative percentages can be taken as no significant contribution of acetogenically formed acetate.

a

b

c

d

Fig. S1 The time course CH_4_ concentration of incubated sediments for (a) control assay of core (I); (b) inhibited assay of core (I); (c) control assay of core (II); (d) inhibited assay of core (II); average ± SE; n = 3.

a

b

c

d

Fig. S2 The time course CO_2_ concentration of incubated sediments for (a) control assay of core (I); (b) inhibited assay of core (I); (c) control assay of core (II); (d) inhibited assay of core (II); average ± SE; n = 3.

Fig. S3 The time course δ^13^C of produced CH_4_ for (a) control assay of core (I); (b) inhibited assay of core (I); (c) control assay of core (II); (d) inhibited assay of core (II); average ± SE; n = 3.

a

b

c

d

Fig. S4 The time course δ^13^C of produced CO_2_ for (a) control assay of core (I); (b) inhibited assay of core (I); (c) control assay of core (II); (d) inhibited assay of core (II); average ± SE; n = 3.

Part of hydrogenotrophic methanogenesis

d

a

Part of hydrogenotrophic methanogenesis

b

e

Part of hydrogenotrophic methanogenesis

c

Fig. S5 Calculated contribution of hydrogenotrophic methanogenesis using different literature values of the fractionation factors (alpha) for acetoclastic methanogenesis. a) 0-10cm; b) 10-20cm, c) 30-40cm, d) 40-50cm, e) 50-60cm.

Fig. S6 Relative contribution of hydrogenotrophic methanogenesis to the released methane of the depth profile of core I if the isotopic signal of the soil organic carbon is used for calculations. Calculated assuming a fractionation factor of α_app_ = 1.009 for acetoclastically produced methane. The 20-30 cm depth did not release methane under inhibited conditions; hence the contribution of hydrogenotrophic methanogens could not be calculated for that sample.

In Fig. 4 we used the measured acetate values to calculate the contribution of hydrogenotrophic methanogenesis. It is however more correct to use the isotopic signal of the acetate-methyl group (which is the precursor of the released methane) instead of total acetate. It is technically difficult to analyze the isotopic composition of the methyl group of acetate. However it has been shown that it is generally -10‰ lighter than the total acetate (Conrad et al. 2014). In our study the signals of the total acetate were already very light (-31.0 to -47.8 ‰) which seemed to justify the use of total acetate.

Alternatively the isotopic values of the soil organic carbon (-25.8 to -26.8 ‰) can be used to estimate the total acetate signal (δ_acetate_ = δ_org soil_ ±10‰) (Conrad et al. 2014). If we assume no fractionation between soil organic carbon and acetate and correct for the depletion of the methyl-group, the δ_acetate-methyl_ value is -35.8 to -36.8‰. This is in the range of the total acetate values (-31.0 to -47.8 ‰) we used for calculation. In general the contribution of hydrogenotrophic methanogenesis is therefore similar if we use the total measured acetate values (Fig 4) or the soil organic carbon derived isotope values (Fig. S6) for the calculations.

Fig. S7 The time course CH_4_ concentration of aerobically incubated sediments with CH_4_ amended headspace to 1% of volume for core (I); average ± SE; n = 3.

In order to determine the CH_4_ oxidation potential (methanotrophy) of river sediment samples, the sediments of the core (I) were incubated in triplicates under wet aerobic conditions: 2 g of wet sediment samples were placed in a 25-ml pressure tube, closed with butyl rubber stoppers, supplemented with 1% (v/v of the headspace) methane, and incubated aerobically at 25°C. Gas subsamples (0.1 – 0.4 ml) were taken repeatedly each 48 hours from the headspace using gas-tight syringe (VICI) and analyzed for concentration of CH_4_. The fastes methane depletion was in the top layer (0-10 cm) (estimated to be 1µmol CH_4_ g^-1^ Sediment day^-1^) and the second fastes in layer 10-20 cm (estimated to be 0.25µmol CH_4_ g^-1^ Sediment day^-1^), other layers proved no CH_4_ depletion during 312 hours incubation (Fig. S6). Based on our results we suppose that methanotrophic activity significantly occures only in the top layer of the sediment.

**Figure S8**. T-RFLP pattern of methanogenic community based on the *mcrA* gene. The individual peaks have been assigned using a clone library (Fig. S9) bp 255, 394, 406, 419, 427, 472-474, 491, and existing literature data 403, 470, 503 bp ([Lueders et al., 2001](#_ENREF_6);[Chin et al., 2004](#_ENREF_3)). The T-RF 269/270 could not be assigned to any published mcrA T-RF, and was not present in our clone library.


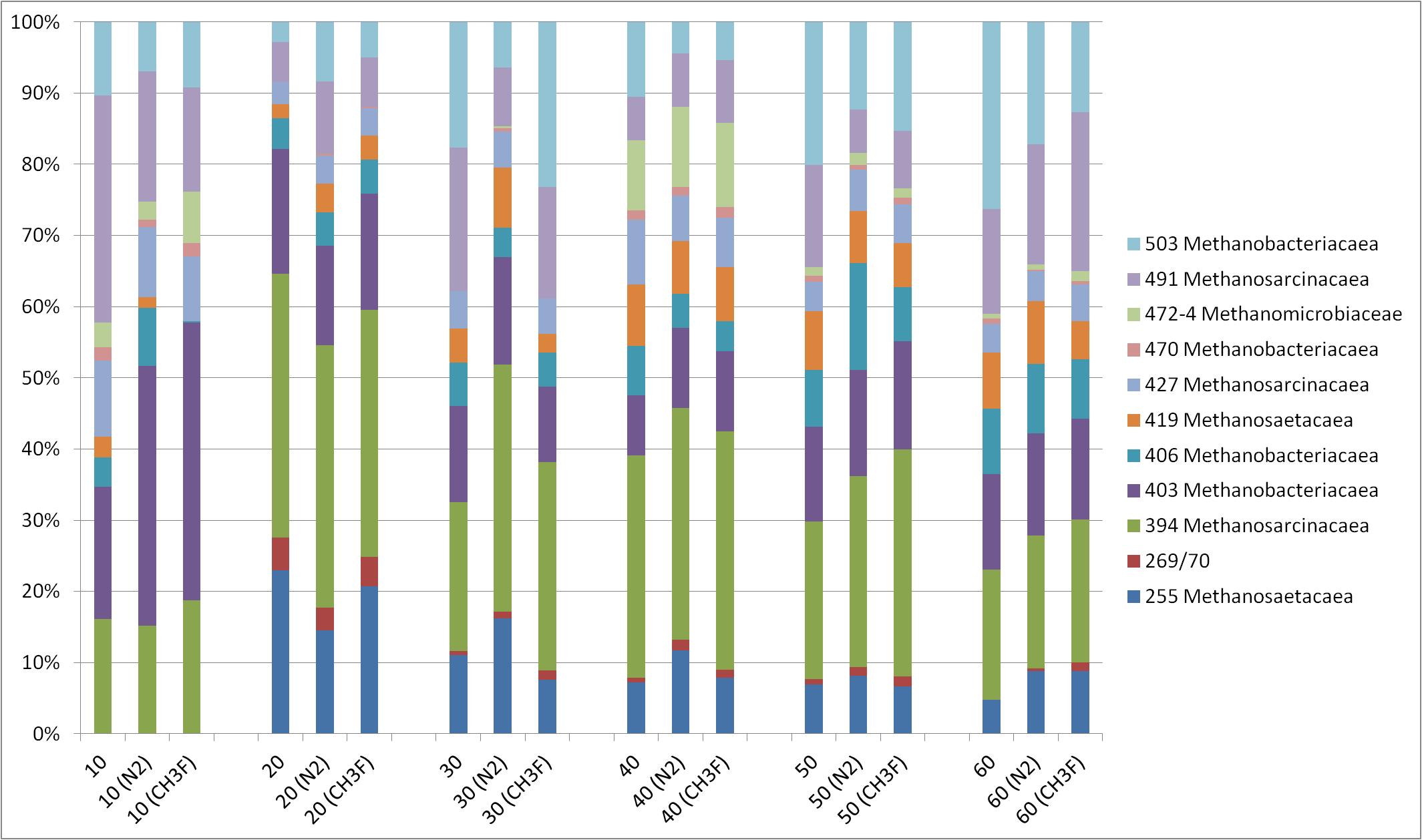


**Figure S9**. Phylogenetic tree of methanogens including clones from our clone library. Each clone is given with it’s accession number and and the in-silico cutting site of the restriction enzym SAU96 used for the T-RFLP analysis. Uncutted fragment are given with the sequence lenght instead. From the 53 clones uesed in the library 57% (30 clones) cluster within the Methanosarcinales 40% (21 clones) withnin the Methanomicrobiales and only 3% (2 clones) within the Methanobacteriales. Methanopyrus kandleri was used as an outgroup to root the tree.
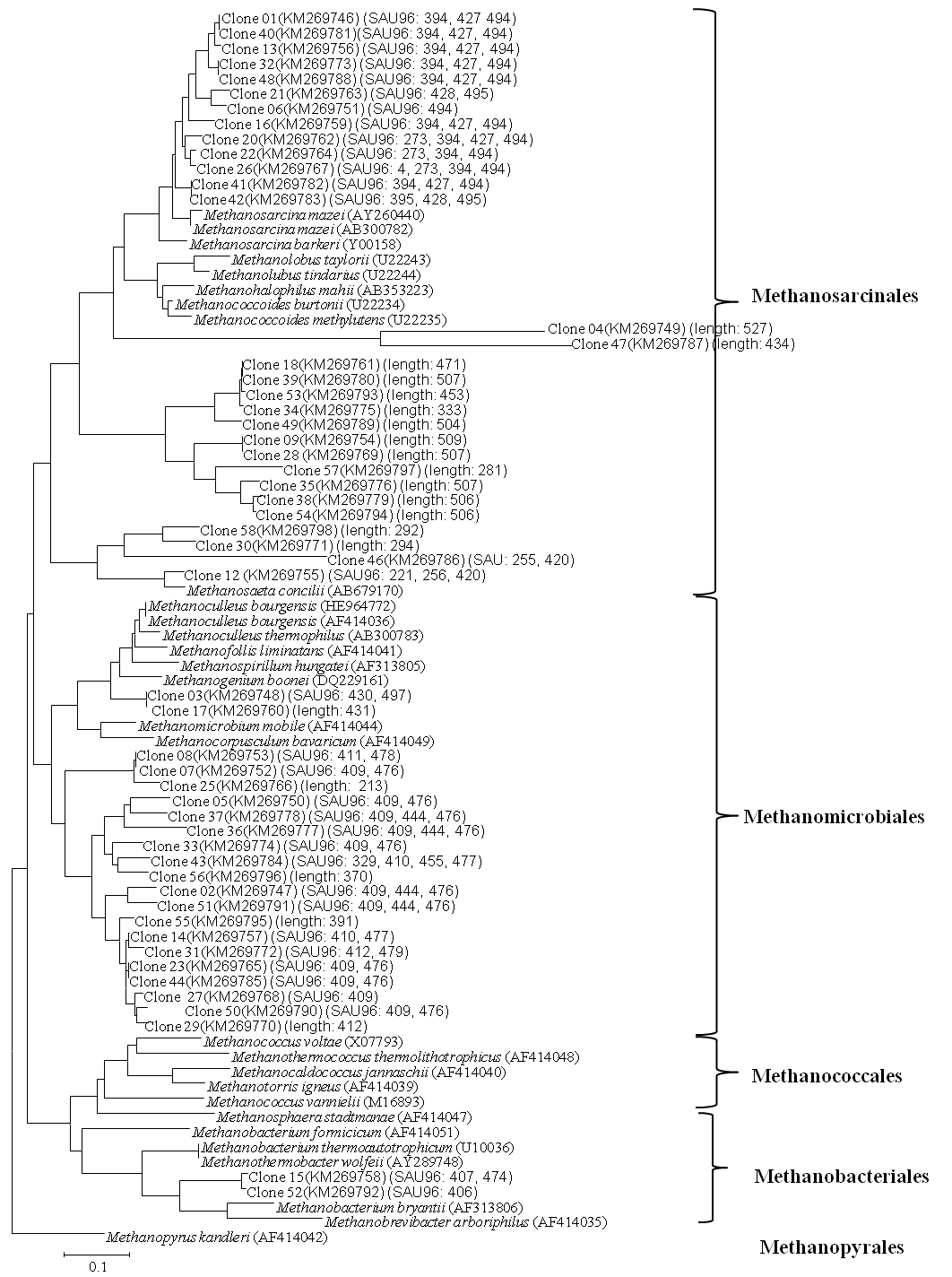


**References**

Blair, N., Leu, A., Munoz, E., Olsen, J., Kwong, E., and Des Marais, D.J. (1985). Carbon isotopic fractionation in heterotrophic microbial-metabolism. *Applied and Environmental Microbiology* 50**,** 996-1001.

Blaser, M.B., Dreisbach, L.K., and Conrad, R. (2013). Carbon isotope fractionation of 11 acetogenic strains grown on H_2_ and CO_2_. *Applied and Environmental Microbiology* 79**,** 1787-1794.

Chin, K.J., Lueders, T., Friedrich, M.W., Klose, M., and Conrad, R. (2004). Archaeal community structure and pathway of methane formation on rice roots. *Microb Ecol* 47**,** 59-67. doi: 10.1007/s00248-003-2014-7.

Conrad, R., Noll, M., Claus, P., Klose, M., Bastos, W., and Enrich-Prast, A. (2011). Stable carbon isotope discrimination and microbiology of methane formation in tropical anoxic lake sediments. *Biogeosciences* 8**,** 795-814.

Gelwicks, J.T., Risatti, J.B., and Hayes, J.M. (1989). Carbon isotope effects associated with autotrophic acetogenesis. *Organic Geochemistry* 14**,** 441-446.

Lueders, T., Chin, K.J., Conrad, R., and Friedrich, M. (2001). Molecular analyses of methyl-coenzyme M reductase alpha-subunit (*mcrA*) genes in rice field soil and enrichment cultures reveal the methanogenic phenotype of a novel archaeal lineage. *Environmental Microbiology* 3**,** 194-204.

Penning, H., and Conrad, R. (2006). Carbon isotope effects associated with mixed-acid fermentation of saccharides by *Clostridium papyrosolvens*. *Geochimica et Cosmochimica Acta* 70**,** 2283-2297.
